# Enhanced recovery after surgery principles in pediatric fractures and sports injuries: strategies, technologies, and outcomes

**DOI:** 10.3389/fped.2026.1815152

**Published:** 2026-07-23

**Authors:** Kang Liu, Lei Yu, Zhongshuo Lu

**Affiliations:** 1 Department of Rehabilitation Medicine, The 960th Hospital of the PLA Joint Logistics Support Force, Jinan, China; 2 Department of Special Service, The 960th Hospital of the PLA Joint Logistics Support Force, Jinan, China

**Keywords:** enhanced recovery after surgery, fractures, multimodal analgesia, pediatrics, perioperative management, rehabilitation outcomes, sports injuries

## Abstract

Enhanced Recovery After Surgery (ERAS) protocols have been widely validated across adult surgical specialties, with consistent evidence showing their ability to improve patient outcomes and shorten recovery durations. In contrast, the application of ERAS principles in the management of pediatric fractures and sports-related injuries is still in the early stages of development; although the supporting evidence remains limited, it is gradually accumulating. This review is designed to systematically summarize the current status of ERAS implementation in the perioperative care of pediatric fractures—especially common injuries such as hand fractures—and sports-related trauma. By integrating epidemiological data regarding the unique characteristics of these injuries in children, this review explores the effective integration of core ERAS components, including multimodal analgesia, minimally invasive surgical approaches, early mobilization, and active family engagement, into pediatric trauma care pathways. Synthesis of the available clinical evidence highlights that ERAS protocols hold considerable potential for optimizing clinical outcomes, accelerating rehabilitation progress, improving the quality of life of young patients, and reducing healthcare expenditures. Despite the promising preliminary findings, several challenges persist, particularly in the standardization of ERAS protocols specifically tailored to the pediatric population. This underscores the necessity for further in-depth research and multidisciplinary collaboration in this field. Overall, this review provides a comprehensive overview of the current strategies, technical considerations, and clinical outcomes associated with ERAS application in pediatric orthopedic trauma, with the goal of guiding clinical practice and future research in this rapidly evolving area.

## 1 Introduction

Children's fractures and sports-related injuries represent a significant proportion of emergency department presentations worldwide, posing unique epidemiological and clinical management challenges (1, 2). Epidemiological data reveal that pediatric fractures commonly involve the upper and lower extremities, with distinct patterns influenced by age, sex, and activity type (3). For instance, hand fractures constitute the second most frequent fracture type in children, with a predominance in males and a peak incidence in school-aged children and adolescents (4–6). The proximal phalanges are most commonly affected, and injury mechanisms vary with age, ranging from entrapment injuries in younger children to ball sports in older children (4). Similarly, distal radius fractures are prevalent, accounting for over a quarter of pediatric fractures, with a male predominance and a higher incidence during spring months, often resulting from high-energy falls or sports participation (7, 8). Physeal fractures tend to occur in older children, while torus and bicortical fractures are more common in younger age groups (7).

Lower extremity fractures, including tibial and femoral fractures, also contribute substantially to pediatric trauma, with sports activities and falls being leading causes (9–11). Notably, tibial shaft fractures have been linked to board sports in certain regions, and femoral fractures show seasonal variation and are often associated with road traffic accidents and sports injuries depending on age (12, 13). The epidemiology of spinal fractures in children has evolved, with a noted decrease in road traffic incidents and an increase in sports-related injuries, predominantly affecting the thoracic and lumbar spine, and with a relatively low incidence of neurological deficits (14, 15). Facial fractures in children, although less frequently than in adults, demonstrate a male predominance and are often sports-related, with variations in fracture types and associated injuries influenced by age and sex (16).

The coronavirus disease 2019 (COVID-19) pandemic has further influenced pediatric trauma epidemiology, with reports indicating an overall decrease in fracture incidence but an increase in certain injury types such as high-acuity assaults and physical abuse, underscoring the complex interplay between societal factors and injury patterns (17–19). Together, these epidemiological insights support the need for tailored clinical approaches that consider injury mechanisms, anatomical characteristics, developmental stages, and recovery-related needs in children.

Traditional management of pediatric fractures and sports injuries has largely focused on stabilization and healing, often emphasizing operative or conservative treatment modalities without comprehensive integration of perioperative optimization strategies (20). However, this treatment-oriented model may not fully address pain control, psychological stress, family participation, and functional recovery, all of which are particularly relevant to children because of their ongoing growth, development, and dependence on caregiver support. Enhanced Recovery After Surgery (ERAS) protocols, initially developed and validated mainly in adult surgical populations, encompass evidence-based perioperative interventions aimed at minimizing surgical stress, preserving physiological function, and expediting recovery. In pediatric trauma care, ERAS principles provide a promising but still incompletely standardized framework that incorporates multimodal analgesia, early mobilization, nutritional optimization, and psychological support tailored to children's specific needs (21).

For example, evidence from pediatric orthopedic trauma and related perioperative settings suggests that multimodal pain management strategies, including regional anesthesia and selected adjuncts such as dexmedetomidine, may reduce opioid exposure and improve postoperative recovery profiles (22). Moreover, psychological factors such as anxiety and depression have been shown to adversely affect rehabilitation outcomes in children undergoing procedures like anterior cruciate ligament reconstruction, emphasizing the importance of holistic care models that address both physical and mental health (23, 24). The unique physiological and psychosocial characteristics of children necessitate adaptation of ERAS protocols to accommodate growth, developmental stages, and family dynamics, ensuring interventions are age-appropriate and family-centered (25–27). Additionally, advanced rehabilitation technologies have shown benefits in selected pediatric rehabilitation settings, although their direct applicability to pediatric fracture and sports injury recovery requires further validation (28).

Despite these advances, the implementation of ERAS in pediatric trauma remains limited, with a paucity of standardized guidelines and consensus on optimal protocols (26, 29). This gap underscores the need for more rigorous and quantitatively informed evaluation of ERAS strategies in pediatric fracture and sports injury populations, particularly with respect to pain scores, opioid consumption, postoperative complications, length of hospital stay, functional milestones, patient- and family-reported outcomes, and healthcare costs.

The incorporation of ERAS protocols into the management of pediatric fractures and sports injuries requires careful consideration of the distinct epidemiological patterns, injury mechanisms, and developmental factors unique to this population. Children present with a diverse spectrum of injuries influenced by age-related activities, such as sports participation, falls, and recreational activities, which necessitate individualized perioperative care plans (30, 31). For instance, the high incidence of sports-related clavicular fractures in adolescents, predominantly males aged 10–14 years, occurring in recreational settings, highlights the importance of targeted prevention and rehabilitation strategies (32). Similarly, the prevalence of hand and foot fractures with associated growth plate injuries in preschool and school-aged children calls for meticulous surgical and rehabilitative approaches to prevent long-term functional impairment (6). In this context, ERAS-oriented pathways should integrate multimodal analgesia to address acute pain while minimizing opioid exposure, early nutritional support to promote healing, and psychological interventions to mitigate anxiety and depression that may hinder recovery (22, 23).

Family involvement and education are also critical components, because the home environment and caregiver support can influence adherence to rehabilitation, recognition of complications, and prevention of re-injury. Technology-supported strategies, including radiation-sparing imaging, telemedicine, remote monitoring, and rehabilitation-assistive tools, may further support ERAS principles by improving safety, follow-up continuity, and patient engagement (33). Furthermore, the COVID-19 pandemic has underscored the need for adaptable ERAS pathways that accommodate changes in injury patterns and healthcare delivery, including telemedicine and remote monitoring to support early discharge and outpatient rehabilitation (17, 18). The development of pediatric-specific ERAS guidelines will require multidisciplinary collaboration among orthopedic surgeons, anesthesiologists, rehabilitation specialists, psychologists, nurses, and caregivers to ensure comprehensive and feasible care.

Outcome measures should extend beyond traditional surgical endpoints to include quantifiable recovery indicators, patient-reported outcomes, psychological well-being, functional status, and family satisfaction, thereby facilitating a more comprehensive assessment of recovery. Ultimately, this review aims to synthesize the available evidence on ERAS strategies for pediatric fractures and sports-related injuries, while critically distinguishing direct pediatric evidence from indirect extrapolation and emphasizing clinically meaningful outcomes such as pain control, opioid exposure, complications, hospitalization duration, functional recovery, family satisfaction, and healthcare-resource care.

## 2 Epidemiological characteristics of pediatric fractures and sports injuries and the rationale of ERAS application

**Figure 1:**
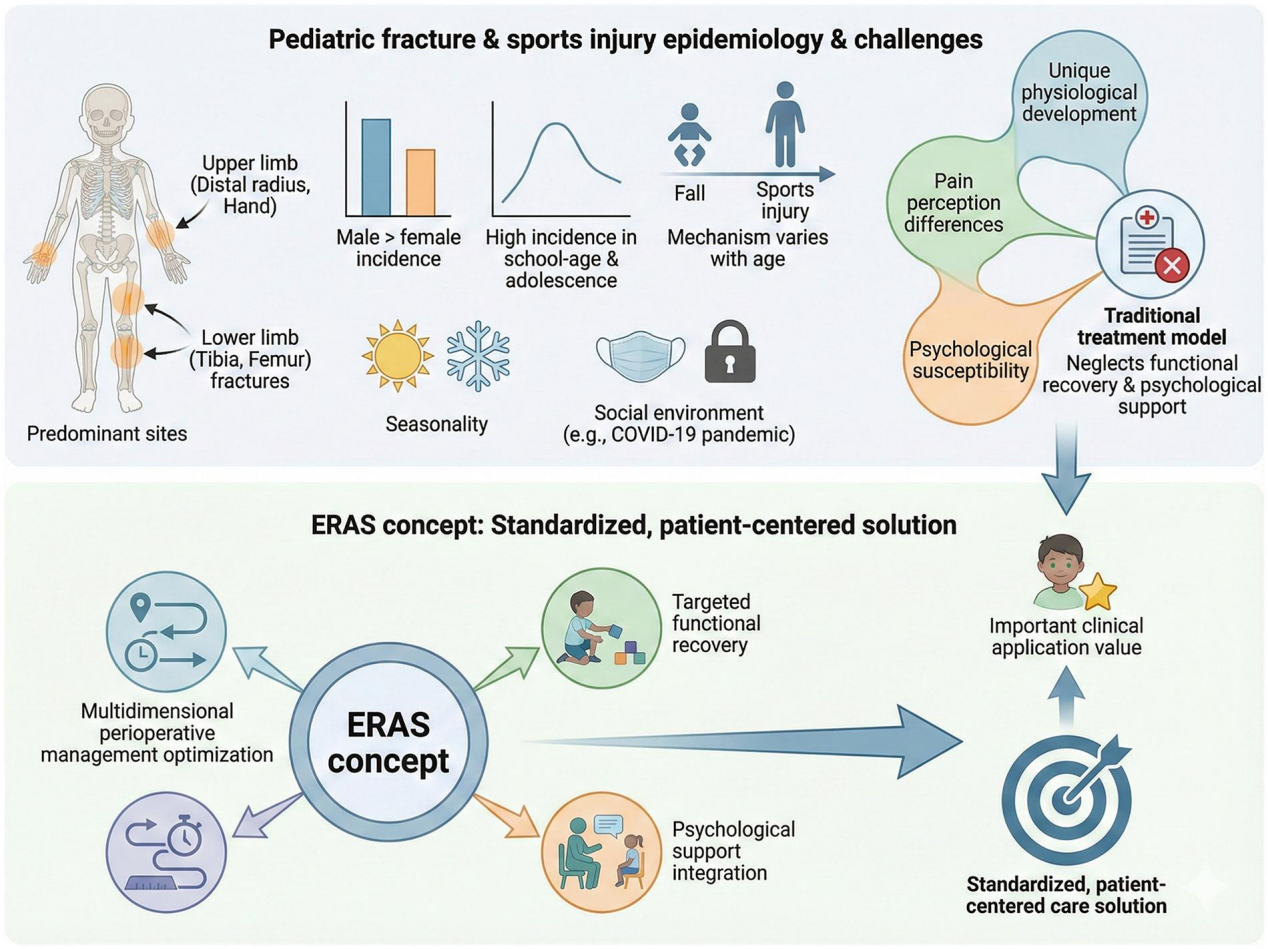
Overview of pediatric fracture and sports injury epidemiology, challenges, and the ERAS solution.Key epidemiological features include predominant injury sites (upper limb: distal radius and hand; lower limb: tibia and femur), a higher incidence in males than females, peak incidence in school-age children and adolescents, age-dependent injury mechanisms (e.g., falls in younger children, sports injuries in adolescents), and influences of seasonality and social environment (e.g., the COVID-19 pandemic). Unique challenges in pediatric trauma management stem from children's distinct physiological development, pain perception differences, and psychological susceptibility, while traditional treatment models often neglect functional recovery and psychological support. The ERAS concept provides a standardized, patient-centered framework that integrates multidimensional perioperative management optimization, targeted functional recovery, and psychological support integration, offering important clinical application value to address these challenges.

Pediatric fractures and sports-related injuries are a major public health concern worldwide, imposing substantial physical, psychological, and economic burdens on children, their families, and healthcare systems. A comprehensive understanding of the epidemiological features, including incidence, risk factors, and injury patterns, is essential for developing targeted prevention strategies and optimizing clinical management. Meanwhile, the unique physiological, developmental, and psychosocial characteristics of children pose considerable challenges to trauma care, highlighting the need for evidence-based and family-centered approaches to improve recovery outcomes. Although ERAS pathways have been widely adopted in adult orthopedic surgery, their application in pediatric orthopedic trauma remains less standardized and requires careful adaptation to children's developmental and family-care contexts. These multifaceted challenges, alongside the evolving landscape of injury epidemiology, provide a strong rationale for integrating ERAS-oriented concepts into pediatric trauma care, as visually summarized in Figure 1.

This section first summarizes the epidemiology and risk factors of common pediatric fractures and sports injuries, then discusses the specific challenges in pediatric trauma management and the rationale for introducing ERAS-oriented pathways into this clinical setting.

### 2.1 Epidemiology and risk factors of common injury types

Large-scale database studies, such as those utilizing the National Electronic Injury Surveillance System (NEISS) and national fracture registries, have consistently demonstrated that pediatric hand fractures represent a significant proportion of childhood injuries, with a notably high incidence in the 10–14 year age group (4, 34–36). Males are disproportionately affected compared to females, with male-to-female ratios ranging from approximately 1.8:1 to over 2:1 across various studies (5, 37, 38). The predominant mechanisms of injury are sports-related activities and falls, which together account for a substantial proportion of pediatric fractures. For example, falls are the leading cause in younger children, while sports injuries become more prevalent in adolescents, particularly males engaged in competitive or recreational sports (39–41). The distribution of fracture types and locations varies with age, sex, and anatomical site. Distal radius and hand phalanx fractures are among the most common, with torus and Salter-Harris fractures frequently observed in younger children (5, 42).

Notably, the risk of sports-related injuries, including fractures, increases during adolescence, with males participating in high-impact and contact sports at greater risk (43, 44). The epidemiology also reveals that specific fracture patterns, such as supracondylar humerus fractures, have distinct age and sex distributions, with boys showing bimodal peaks in early childhood and late adolescence (45, 46). Furthermore, the COVID-19 pandemic has significantly altered injury epidemiology, with an overall decrease in fracture incidence due to reduced sports participation and outdoor activities, but a relative increase in injuries occurring at home or during bicycling (47–49). These shifts underscore the dynamic nature of pediatric injury epidemiology and the need for adaptable prevention, triage, perioperative management, and rehabilitation strategies. Socioeconomic status also influences fracture incidence, with children in higher socioeconomic regions exhibiting increased fracture rates, possibly due to greater sports participation and activity levels (50, 51).

Collectively, these epidemiological insights indicate that pediatric fractures are multifactorial in origin, with age, sex, activity type, socioeconomic context, and environmental factors jointly shaping injury risk profiles.

### 2.2 Unique challenges in pediatric trauma management and the value of ERAS implementation

Pediatric trauma management presents unique challenges distinct from adult care, primarily due to the physiological, psychological, and developmental characteristics of children (52, 53). Children differ from adults in pain expression, communication ability, and developmental understanding of illness, which may complicate accurate pain assessment and individualized analgesic management. Postoperative nausea and vomiting (PONV) also require particular attention in pediatric anesthesia, as children have a high incidence of PONV and may be approximately twice as likely as adults to experience it, thereby affecting comfort, oral intake, discharge readiness, and family satisfaction (54–56). Additionally, children frequently experience medical fear and perioperative anxiety, which may reduce cooperation, treatment adherence, and engagement in postoperative rehabilitation. Traditional fracture management often emphasizes anatomical realignment and skeletal stabilization but may insufficiently address functional recovery, psychological distress, return-to-school or return-to-sport concerns, and the burden placed on families (57–60).

The ERAS paradigm offers a structured, multidisciplinary approach for addressing these challenges through coordinated perioperative care, although pediatric fracture-specific protocols remain less mature than adult ERAS pathways (25). Quantitative evidence from pediatric ERAS studies supports the clinical relevance of these components: in a systematic review and meta-analysis including 16 studies and 1,723 children, ERAS implementation reduced the time to initiate feeding by 21.20 h, shortened the time to full enteral nutrition by 2.20 days, reduced postoperative opioid use by 0.86 morphine-equivalent mg/kg, and decreased hospital length of stay by 2.54 days without increasing complications or readmissions (61). Although these data are derived from broader pediatric surgical populations rather than exclusively from fracture surgery, they provide practical quantitative support for core ERAS elements that are clinically relevant to pediatric orthopedic trauma.

In pediatric orthopedics, ERAS-oriented care may mitigate anxiety through child-friendly education and communication, enhance pain control with multimodal strategies, and facilitate earlier mobilization; in a before-after observational study of 4,098 children undergoing pediatric orthopedic surgery, implementation of an interdisciplinary ERAS protocol was associated with a significant difference in hospitalization time and improved financial indicators, including a reduction in the average cost-to-revenue ratio from 82.70% to 72.16% (62–65). Moreover, ERAS facilitates coordinated care involving surgeons, anesthesiologists, nurses, physical therapists, psychologists, and caregivers, thereby supporting a holistic model that addresses both physical recovery and psychosocial well-being (59, 60). The adaptability and flexibility of ERAS protocols are particularly valuable in the context of evolving epidemiological patterns, such as those observed during the COVID-19 pandemic, enabling responsive modifications to care pathways (47).

Overall, ERAS implementation in pediatric trauma care is a promising strategy for shortening recovery time, improving functional outcomes, and reducing psychosocial and economic burdens; however, fracture-specific pediatric evidence remains limited, and future studies should report standardized quantitative outcomes such as pain scores, opioid consumption, PONV incidence, mobilization milestones, length of stay, readmissions, and procedure-specific costs.

## 3 Core strategies of ERAS in perioperative management of pediatric fractures

**Figure 2:**
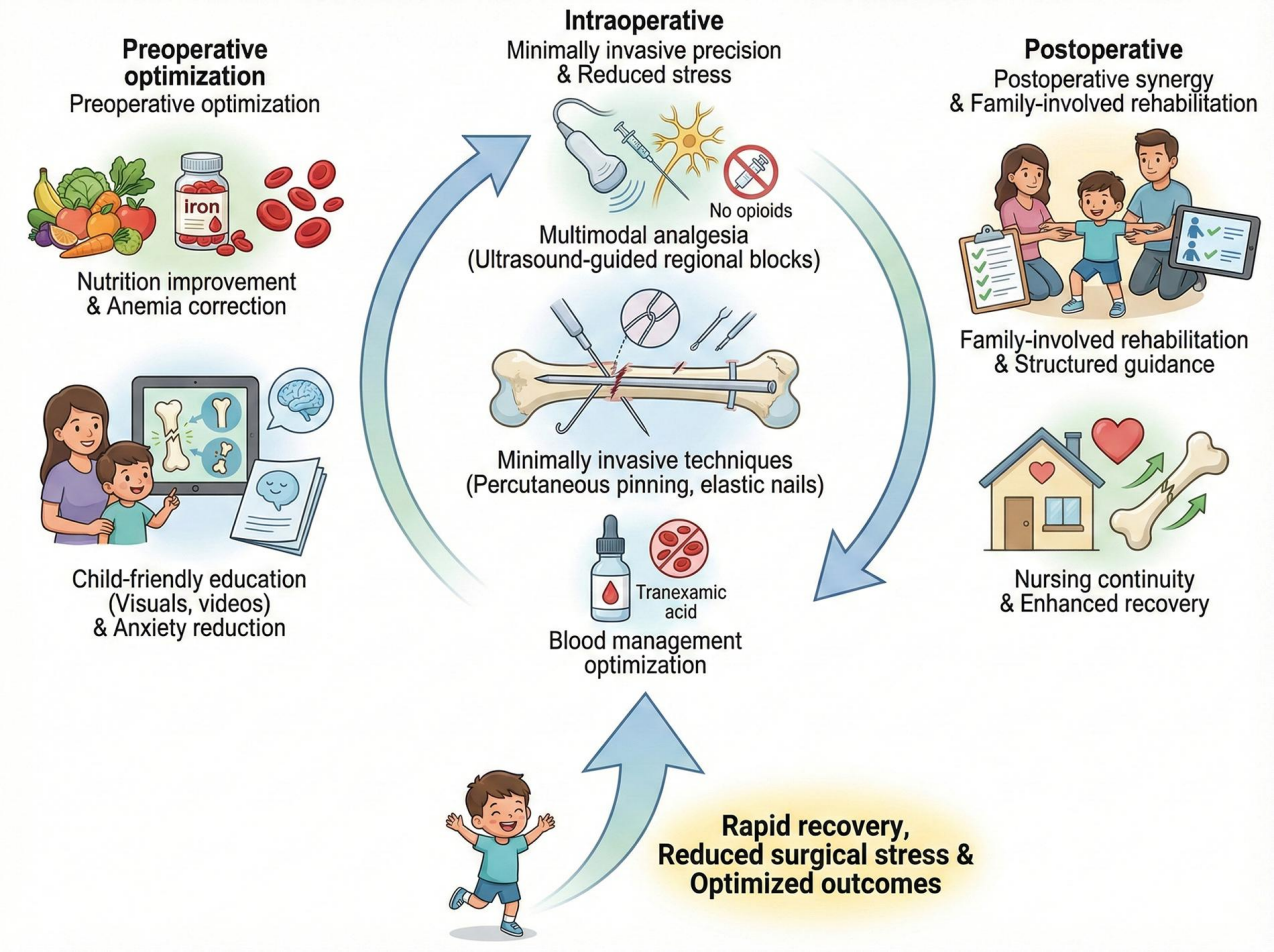
Schematic representation of the ERAS pathway in pediatric fracture care, highlighting key perioperative components. The ERAS framework is organized into three interconnected phases: (1) Preoperative optimization, including nutrition improvement, anemia correction, child-friendly education, and anxiety reduction; (2) Intraoperative management, featuring minimally invasive surgical techniques (e.g., percutaneous pinning, elastic nails), multimodal analgesia (ultrasound-guided regional blocks with opioid avoidance), and blood management optimization (e.g., tranexamic acid); and (3) Postoperative synergy, emphasizing family-involved rehabilitation with structured guidance and nursing continuity to enhance recovery. This integrated approach aims to achieve rapid recovery, reduce surgical stress, and optimize overall clinical outcomes for children with fractures.

The application of ERAS principles in the perioperative management of pediatric fractures is intended to mitigate surgical stress, reduce perioperative complications, accelerate functional recovery, and improve the overall recovery experience of children. Unlike adults, children have unique physiological, psychological, developmental, and family-care needs, which require ERAS protocols to be adapted rather than directly transferred from adult orthopedic practice. Conceptually, the ERAS pathway in pediatric fracture care can be organized into a three-phase perioperative framework encompassing preoperative optimization, intraoperative minimally invasive and analgesic strategies, and postoperative family-centered rehabilitation, as illustrated in Figure 2. This section focuses on core ERAS-oriented strategies in pediatric fracture care, including preoperative optimization, multimodal analgesia, minimally invasive fixation, blood management, and supportive perioperative measures, with emphasis on available clinical evidence and quantitative outcomes where reported.

### 3.1 Preoperative optimization and education

Preoperative optimization and education are important components of ERAS-oriented care for pediatric orthopedic trauma, including fractures and sports injuries (63, 66). A comprehensive preoperative assessment of the child and family is essential, including nutritional status, anemia risk, baseline pain and anxiety, caregiver understanding, and anticipated barriers to postoperative rehabilitation (67). Nutritional optimization is clinically relevant because malnutrition, inadequate protein-energy intake, vitamin D deficiency, and low bone mineral density may compromise postoperative recovery and skeletal health in children undergoing orthopedic procedures (68). For children at high risk of perioperative blood loss, preoperative anemia screening and patient blood management should be considered, because pediatric evidence indicates that preoperative anemia is associated with increased transfusion risk and prolonged hospital stay in major orthopedic procedures such as spine arthrodesis (69, 70).

Psychological preparation tailored to the child's developmental level, including age-appropriate tools such as animated picture books, videos, and structured explanations, can reduce preoperative anxiety and improve cooperation during perioperative care (71, 72). Implementing family-centered care models can integrate parents or caregivers into the rehabilitation team, equipping them with basic skills for pain observation, limb protection, home exercise supervision, and early recognition of warning signs (73). For elective surgeries related to sports injuries, such as anterior cruciate ligament reconstruction, individualized prehabilitation aimed at maintaining muscle strength, neuromuscular control, and joint range of motion may improve postoperative functional recovery; however, this evidence should be interpreted as sports-surgery evidence rather than fracture-specific ERAS evidence (74). Preoperative education also addresses expectations regarding pain management, opioid use, and functional recovery, which is crucial given the documented impact of psychological distress on postoperative outcomes (75).

More specifically, a narrative review of preoperative patient education identified 34 eligible studies; among them, all 8 studies assessing postoperative opioid use reported reduced opioid consumption after education, and 5 of 12 studies assessing postoperative pain reported pain reduction (71). Collectively, these elements support a multidisciplinary and individualized approach to preoperative optimization in pediatric orthopedic ERAS pathways, while also underscoring the need for future pediatric fracture-specific studies that quantify outcomes such as anxiety scores, opioid use, early mobilization, complications, and length of stay.

### 3.2 Multimodal analgesia and anesthesia management

Multimodal analgesia and anesthesia management are central components of ERAS protocols designed to reduce opioid exposure, improve pain control, and facilitate early mobilization in pediatric orthopedic surgery (62). Regional nerve blocks, such as ultrasound-guided brachial plexus blocks for upper limb fractures, can provide effective opioid-sparing analgesia when performed by experienced clinicians; for example, in a matched cohort study of children younger than 12 years undergoing elbow fracture surgery, supraclavicular brachial plexus block reduced total opioid consumption to the first postoperative day from 3.2 ± 3.0 mg to 0.9 ± 1.8 mg compared with general anesthesia alone (76). The integration of acetaminophen and nonsteroidal anti-inflammatory drugs (NSAIDs) can form the non-opioid foundation of multimodal analgesia, although dosing should be individualized according to age, weight, renal function, bleeding risk, and surgical context (77).

In pediatric fracture care, ibuprofen has been shown to provide analgesia comparable to acetaminophen with codeine for arm fractures, with fewer adverse effects and better functional outcomes; importantly, available pediatric evidence does not suggest that short-term ibuprofen use impairs clinical or radiographic long-bone fracture healing (78, 79). Attention to opioid-related adverse effects, including nausea, vomiting, oversedation, constipation, and potential misuse, supports opioid stewardship, prophylactic antiemetic strategies in high-risk children, and clear discharge instructions regarding opioid duration and tapering (80). Pediatric fracture-specific studies also support opioid-sparing strategies: after closed reduction and percutaneous pinning for supracondylar humeral fractures, pain and opioid use decreased to clinically minor levels by postoperative day 3, and children used less than 25% of the opioids prescribed after discharge (81).

Similarly, perioperative ketorolac use in children with supracondylar humerus fractures has been reported to decrease postoperative pain, opioid use, hospitalization cost, and length of stay, further supporting the role of NSAID-based opioid-sparing regimens in selected pediatric fracture patients (82). Furthermore, preoperative education about pain management strategies enhances patient and family understanding, contributing to better adherence and outcomes (71). Taken together, regional anesthesia combined with systemic multimodal analgesics represents a clinically supported strategy to improve perioperative pain control, reduce opioid exposure, and facilitate early mobilization in children undergoing fracture fixation or sports injury surgery, although the optimal regimen should be individualized and remains insufficiently standardized across pediatric fracture populations.

### 3.3 Minimally invasive surgical techniques and blood management

Minimally invasive fixation techniques and meticulous blood management are important ERAS-oriented strategies aimed at reducing tissue trauma, limiting blood loss, and facilitating earlier rehabilitation in pediatric orthopedic trauma. Techniques such as closed reduction with percutaneous pinning and elastic stable intramedullary nailing are commonly used in appropriately selected pediatric fractures because they can reduce soft-tissue disruption, preserve growth-related anatomy, and support earlier functional rehabilitation (83, 84). Intraoperative imaging modalities, including C-arm fluoroscopy, can support accurate fracture reduction and fixation, but their use should be balanced with radiation-sparing principles and efforts to avoid unnecessary prolongation of operative and anesthesia time (83). Blood conservation strategies are particularly relevant in pediatric procedures with anticipated blood loss. Tranexamic acid (TXA) may reduce intraoperative bleeding and transfusion requirements, but the current pediatric orthopedic evidence is derived mainly from elective orthopedic procedures and children with cerebral palsy rather than acute fracture fixation (85).

Therefore, TXA should be discussed as a potentially useful blood-management tool for selected high-risk pediatric orthopedic trauma cases, rather than as a universally required component of pediatric fracture ERAS protocols. Careful surgical technique, meticulous hemostasis, normothermia, and avoidance of unnecessary tissue dissection may further contribute to blood conservation. When combined with effective analgesia and early rehabilitation, these approaches may contribute to shorter hospitalization, fewer procedure-related complications, and improved early recovery, although procedure-specific pediatric fracture data remain limited. The adoption of minimally invasive fixation in pediatric orthopedic surgery is consistent with ERAS principles, but its contribution to ERAS outcomes should be evaluated separately from other pathway components such as analgesia, mobilization, nutrition, and discharge planning (83, 84). Additionally, pediatric-tailored blood management protocols may optimize perioperative care by integrating anemia screening, blood-loss risk assessment, antifibrinolytic use when appropriate, restrictive transfusion principles, and postoperative monitoring.

Thus, combining minimally invasive fixation methods with individualized blood conservation measures provides a practical framework for pediatric fracture and sports injury surgery within ERAS-oriented pathways, while future studies should report quantitative endpoints such as operative time, blood loss, transfusion rate, postoperative pain, mobilization timing, length of stay, and cost.

## 4 Early interventions for enhanced recovery after surgery

**Figure 3:**
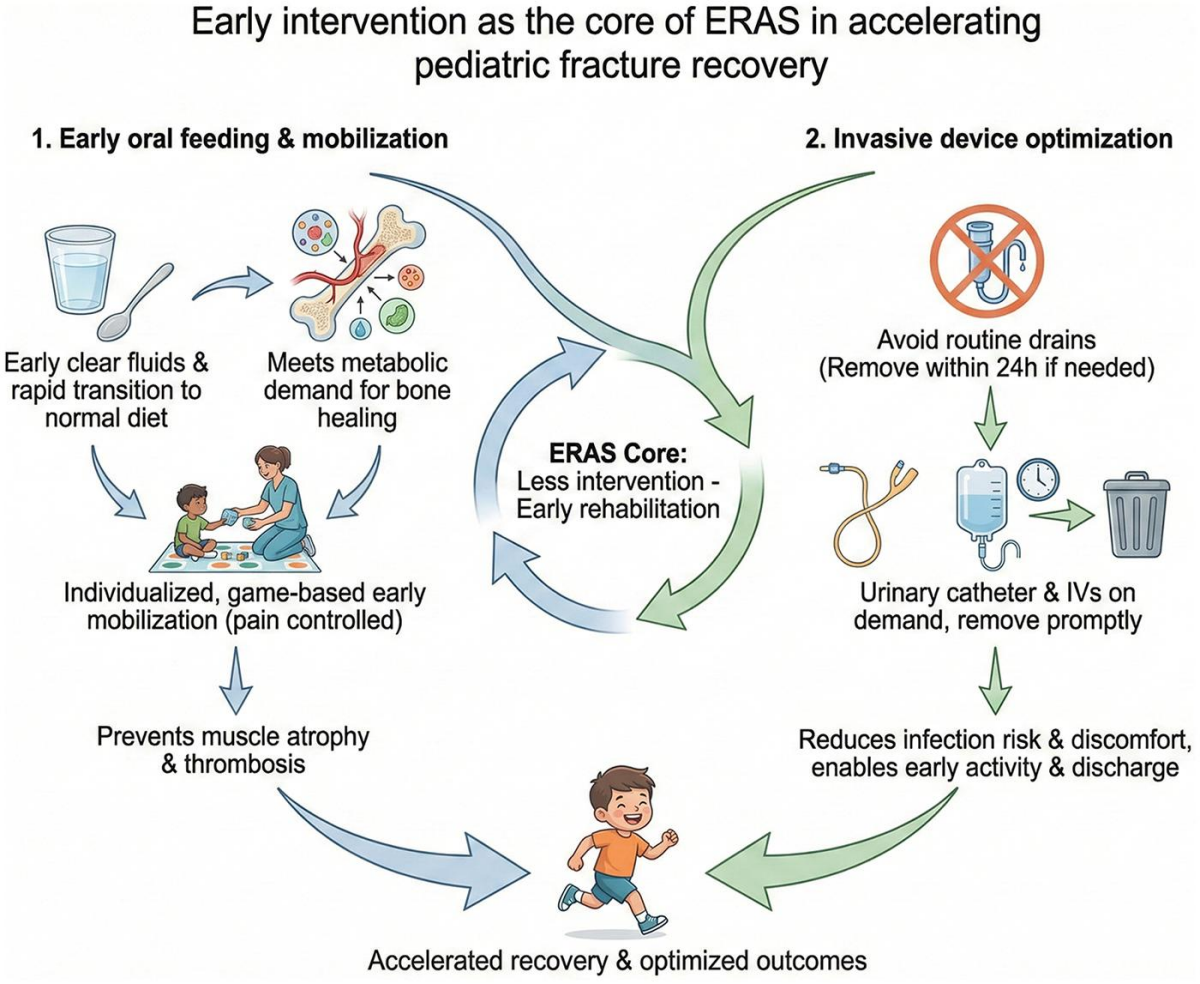
Early intervention as the core of ERAS in accelerating pediatric fracture recovery. The ERAS core principle of “less intervention – early rehabilitation” is implemented through two key strategies: (1) Early oral feeding and mobilization, which involves early clear fluids and rapid transition to a normal diet to meet metabolic demands for bone healing, alongside individualized, game-based early mobilization (pain-controlled) to prevent muscle atrophy and thrombosis; and (2) Invasive device optimization, which entails avoiding routine drains (removal within 24 h if needed) and using urinary catheters and IVs only on demand with prompt removal, thereby reducing infection risk and discomfort and enabling early activity and discharge. Together, these strategies drive accelerated recovery and optimized outcomes for children with fractures.

ERAS protocols represent a multimodal, evidence-based approach to perioperative care that has improved postoperative recovery across multiple surgical specialties. In pediatric orthopedics, ERAS implementation may be particularly meaningful because children have unique physiological, developmental, psychological, and family-care needs, although fracture-specific evidence remains relatively limited. Early postoperative interventions, particularly timely oral intake, safe mobilization, and minimization of unnecessary invasive devices, are practical ERAS components that may influence recovery speed, complications, hospital stay, and functional outcomes. For pediatric fracture care, these strategies should be interpreted as an early-intervention framework rather than a fully standardized protocol, as visually summarized in Figure 3. This section summarizes early oral intake, mobilization, and optimization of invasive perioperative devices, with emphasis on available quantitative evidence and on distinguishing direct pediatric orthopedic evidence from indirect evidence derived from broader pediatric or adult surgical populations.

### 4.1 Early oral intake and mobilization

The shift from prolonged postoperative fasting to early oral intake is consistent with ERAS principles, but in pediatric fracture surgery it should be applied according to anesthesia recovery, aspiration risk, surgical complexity, and the child's tolerance. Quantitative pediatric evidence supports the safety and feasibility of early postoperative hydration: in a randomized controlled trial of 61 children aged 1–5 years undergoing elective surgery, oral hydration within 30 min of post-anesthesia care unit (PACU) arrival resulted in a lower Face, Legs, Activity, Cry, Consolability (FLACC) score at 1 h than delayed hydration after 2 h [1 (0.25–2) vs. 2 (1–3), P = 0.028], with higher parental satisfaction and no major complications (86). Similarly, randomized pediatric anesthesia studies have shown that early oral fluid intake after general anesthesia does not increase clinically important adverse events such as vomiting or aspiration, and may reduce thirst, discomfort, postoperative vomiting, or opioid requirement in selected day-case surgical populations (87–89).

At the ERAS pathway level, a pediatric systematic review and meta-analysis including 16 studies and 1,723 children reported that ERAS reduced the time to initiate feeding by 21.20 h, shortened the time to full enteral nutrition by 2.20 days, reduced postoperative opioid use by 0.86 morphine-equivalent mg/kg, and decreased hospital length of stay by 2.54 days without increasing complications or readmissions (61). However, because most of these data were derived from broader pediatric surgical populations, their applicability to pediatric fracture surgery should be considered supportive rather than definitive. In pediatric orthopedic trauma, early oral intake and adequate nutritional support are physiologically reasonable because bone healing and growth require sufficient energy, protein, and micronutrient availability; nevertheless, direct studies quantifying the effect of early feeding on fracture union or functional recovery in children remain scarce.

Concurrently, early mobilization is a key ERAS component intended to limit deconditioning, support functional recovery, and reduce immobilization-related complications when fracture stability and pain control allow. For children with lower extremity fractures, protected weight-bearing and range-of-motion exercises should be guided by fracture type, fixation stability, weight-bearing restrictions, pain control, and surgeon-directed rehabilitation protocols. Age-appropriate rehabilitation programs, including play-based or task-oriented exercises, may improve engagement and adherence in children; in pediatric tibial eminence fractures, initiation of range-of-motion therapy within 4 weeks after treatment was associated with earlier return to full activity and a lower likelihood of arthrofibrosis (90). Evidence from broader ERAS populations indicates that early mobilization can reduce postoperative complications, improve functional walking recovery, and shorten hospital stay, but the specific timing and measurable benefits in pediatric fracture surgery remain insufficiently defined (91).

Effective pain control through multimodal analgesia and, where appropriate, regional anesthesia can facilitate early mobilization by reducing pain-related avoidance of movement; however, the choice of block should be individualized to avoid masking neurovascular symptoms or impairing safe motor function (92). Importantly, early mobilization protocols must be individualized according to age, injury severity, fixation stability, weight-bearing restrictions, pain control, and family capacity to supervise rehabilitation, with multidisciplinary coordination to ensure safety and maximize functional recovery.

### 4.2 Optimization of drains, urinary catheters, and intravenous access

The management of invasive devices, including surgical drains, urinary catheters, and peripheral intravenous (IV) lines, is an important practical component of pediatric orthopedic ERAS pathways because these devices may contribute to discomfort, infection risk, delayed mobilization, and prolonged hospitalization. Routine drain placement should be avoided when there is no clear clinical indication, because available orthopedic evidence does not support routine closed-suction drainage for all procedures and suggests that drains may increase discomfort, transfusion exposure, or length of stay in some pediatric orthopedic settings (93, 94). When drains are necessary, removal should be considered as soon as clinically safe, based on drain output, wound condition, bleeding risk, and surgeon judgment, rather than being applied as a fixed rule for all pediatric fracture procedures (95). Similarly, urinary catheterization should be avoided in short, uncomplicated pediatric fracture procedures whenever feasible and reserved for clear indications such as prolonged surgery, strict urine-output monitoring, neuraxial anesthesia, or expected postoperative immobility.

When urinary catheters are used, daily reassessment and early removal are consistent with catheter-associated urinary tract infection (CAUTI) prevention principles; catheter-associated urinary tract infection remains a major device-associated infection, and evidence from colorectal ERAS populations suggests that earlier catheter removal can reduce urinary tract infection rates and shorten hospital stay, although these data are indirect for pediatric fracture surgery (96, 97).

Intravenous access should be optimized by using the least invasive and shortest necessary duration compatible with safe hydration, analgesia, antibiotic administration, and emergency access. Early transition from IV fluids and IV analgesia to oral hydration and oral analgesics should be considered once the child is awake, hemodynamically stable, not vomiting, and able to tolerate oral intake; pediatric randomized evidence indicates that early postoperative oral hydration can improve comfort and parental satisfaction without major complications in selected elective surgical patients (86). Removal of peripheral IV lines as soon as clinically feasible may improve comfort and facilitate mobilization, while also reducing catheter-related problems such as infiltration, phlebitis, occlusion, and accidental dislodgement, which are common reasons for peripheral IV catheter failure in children (98, 99).

Collectively, device minimization may support earlier mobilization, improve comfort, and reduce preventable device-related complications, but future pediatric fracture ERAS studies should report device-specific outcomes such as drain use, time to drain removal, urinary catheter duration, IV-line duration, CAUTI, peripheral IV failure, mobilization timing, and length of stay. Multidisciplinary collaboration among surgeons, anesthesiologists, nursing staff, rehabilitation therapists, patients, and caregivers is essential to implement device-management protocols safely, because device removal must be coordinated with pain control, wound assessment, hydration status, mobility goals, and discharge planning.

## 5 Digital health and specialized technologies supporting enhanced recovery after surgery implementation

**Figure 4:**
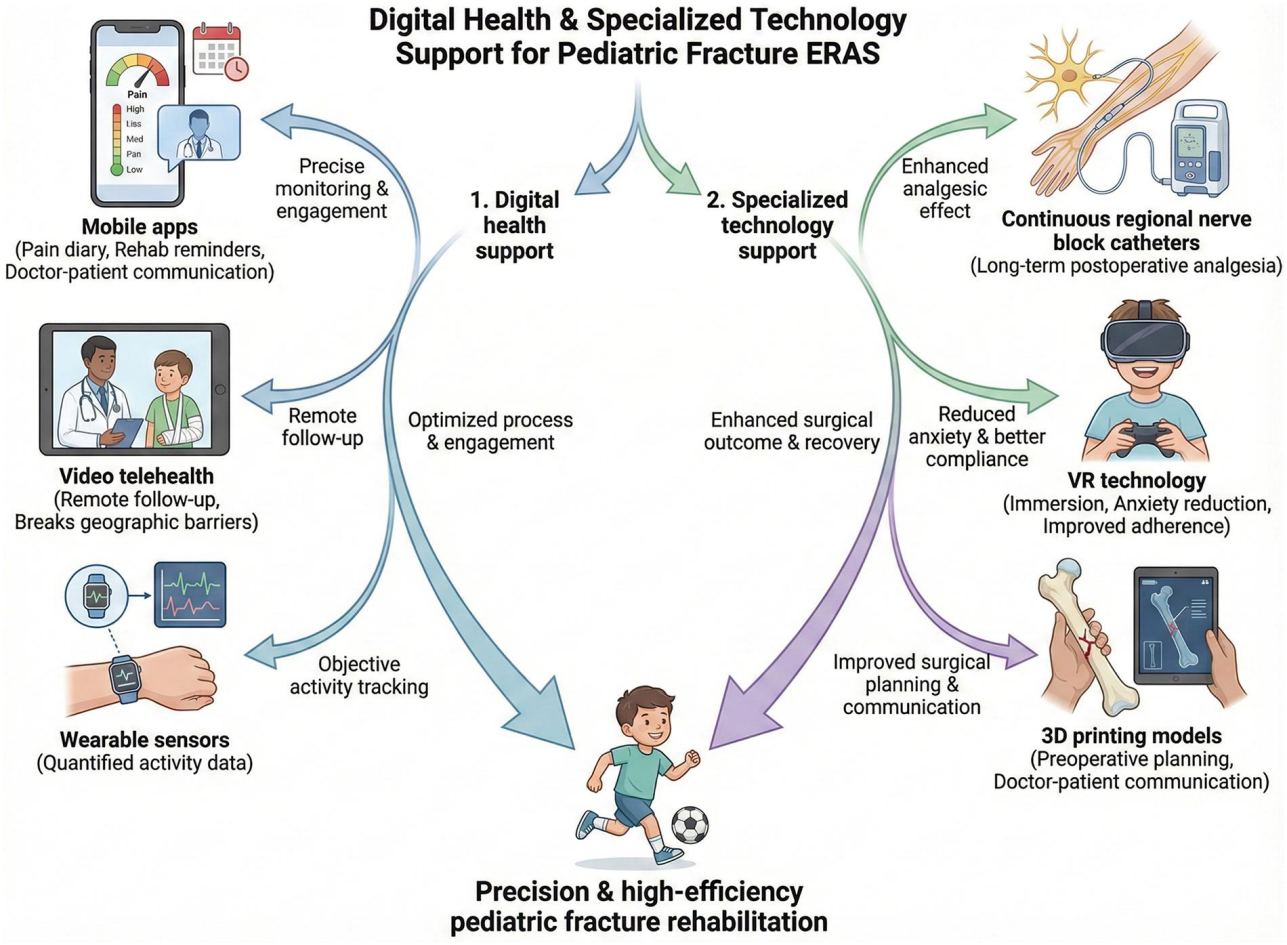
Digital health and specialized technology support for pediatric fracture ERAS, enabling precision and high-efficiency rehabilitation. This framework is structured into two interconnected pillars: (1) Digital health support, including mobile apps for pain diary tracking, rehabilitation reminders, and doctor-patient communication; video telehealth for remote follow-up to break geographic barriers; and wearable sensors for objective activity tracking, all of which enable precise monitoring, optimized processes, and enhanced patient engagement. (2) Specialized technology support, encompassing continuous regional nerve block catheters for long-term postoperative analgesia, VR technology for anxiety reduction and improved adherence, and 3D printing models for enhanced surgical planning and communication. Together, these technologies drive enhanced surgical outcomes, recovery, and ultimately, precision and high-efficiency pediatric fracture rehabilitation.

Digital health and specialized technologies may support ERAS implementation by improving perioperative monitoring, analgesic delivery, rehabilitation guidance, and communication between clinicians, children, and caregivers. In pediatric orthopedic surgery, these tools may be particularly useful because pain reporting, treatment adherence, rehabilitation engagement, and caregiver participation differ substantially from adult care. However, their benefits should be evaluated using measurable endpoints, including pain scores, opioid consumption, visit burden, adherence to rehabilitation, activity levels, complications, family satisfaction, and healthcare costs. The potential roles of digital health and specialized technology in pediatric fracture ERAS are summarized in Figure 4, but the current evidence should be interpreted as supportive rather than definitive because fracture-specific pediatric trials remain limited. This section discusses digital follow-up, mobile applications, wearable monitoring, continuous regional analgesia,virtual reality, and three-dimensional (3D) printing, with emphasis on clinically reported outcomes and the distinction between direct pediatric orthopedic evidence and indirect evidence from broader surgical populations.

### 5.1 Digital health technologies and telemedicine

The integration of digital health technologies and telemedicine into ERAS-oriented care may provide a practical approach to postoperative follow-up, symptom monitoring, rehabilitation supervision, and family education in pediatric orthopedic trauma and sports injuries. Mobile applications can support postoperative monitoring by allowing children and caregivers to record pain, medication use, activity progression, wound-related concerns, and rehabilitation adherence, while also receiving reminders and instructions from the clinical team. Evidence from pediatric postoperative recovery suggests that digital health interventions may improve caregiver knowledge and satisfaction; in a systematic review including 16 studies and 2,728 children, digital interventions significantly improved parental knowledge and satisfaction regarding perioperative instructions, although they did not significantly reduce children's pain intensity (100).

In sports-injury rehabilitation, mobile app-guided self-rehabilitation after anterior cruciate ligament reconstruction has been associated with better early knee function: in one study of 65 patients, app adherence was 91% at 10 days, 71% at 15 days, 62% at 21 days, and 44% at 45 days, and app users were more likely to walk with quadriceps locking and to be pain-free at 3 weeks (101). Although this evidence comes from sports-surgery rehabilitation rather than pediatric fracture surgery, it supports the feasibility of app-based rehabilitation reminders and adherence tracking within ERAS-oriented musculoskeletal recovery pathways. Video-based remote follow-up may be useful for selected stable injuries by allowing clinicians to assess wound appearance, range of motion, functional progress, and caregiver concerns without requiring every child to attend an in-person visit. This approach is especially beneficial for families residing in remote or underserved areas, where travel to specialized centers may be burdensome or impractical.

For pediatric fracture care, telemedicine and virtual fracture clinic models may reduce unnecessary outpatient visits and travel burden; for example, a pediatric virtual fracture clinic systematic review reported that all children were reviewed within 72 h, with limited missed fractures, re-presentation rates comparable to face-to-face clinics, cost savings, and high parent satisfaction (102). In torus fracture management, introduction of a virtual fracture clinic has also been reported to improve efficiency and reduce costs, supporting its potential role for low-risk, protocol-driven pediatric fracture pathways (103). However, telemedicine should not replace in-person assessment when there is concern about neurovascular compromise, worsening pain, wound infection, cast problems, loss of reduction, safeguarding issues, or poor caregiver understanding. Wearable devices equipped with accelerometers and heart-rate sensors can provide objective data on activity level, gait recovery, sleep, and physiological recovery patterns, which may complement subjective pain scores and caregiver reports.

In pediatric postoperative monitoring, wearable-derived data have shown early promise: in a study of children recovering after appendectomy, wearable-derived biorhythm metrics predicted postoperative complications up to 3 days before formal diagnosis with 91% sensitivity and 74% specificity (104). Nevertheless, wearable monitoring in pediatric fracture ERAS should be viewed as a developing adjunct, and future studies should validate activity thresholds, adherence metrics, and alert criteria specifically for orthopedic trauma recovery. More broadly, a systematic review of mobile and wearable technologies after surgery supports their potential for measuring postoperative outcomes, but also emphasizes heterogeneity in devices, outcome definitions, and reporting quality. Collectively, digital health tools may enhance continuity of care and provide measurable recovery data, but their value in pediatric fracture ERAS should be assessed through standardized outcomes such as visit reduction, response time, complication detection, rehabilitation adherence, patient and caregiver satisfaction, and cost.

Therefore, mobile apps, telemedicine, and wearable technology should be presented as supportive ERAS tools that may improve engagement and self-management, rather than as independently proven interventions for improving all clinical outcomes in pediatric fracture patients.

### 5.2 Advanced analgesia and rehabilitation technologies

Advanced analgesic and rehabilitation technologies may complement ERAS-oriented care in pediatric fracture and sports injury surgery by improving pain control, reducing anxiety, supporting rehabilitation engagement, and assisting surgical planning. Continuous peripheral nerve block catheters can provide prolonged, targeted analgesia after selected pediatric orthopedic procedures and may be continued after discharge within structured ambulatory programs (105, 106). Large pediatric audits support feasibility: one audit of 1,285 children reported that ambulatory continuous peripheral nerve blocks can provide postoperative analgesia and may reduce the need for inpatient parenteral opioid therapy, while another pediatric series reported 93.1% ambulatory efficacy (106, 107). Adequate regional analgesia may facilitate participation in early rehabilitation by reducing pain-related avoidance of movement, but clinicians must monitor for motor weakness, catheter malfunction, infection, local anesthetic toxicity, and the possibility of masking evolving neurovascular symptoms.

Thus, ambulatory regional analgesia is most appropriate for carefully selected children and families with reliable education, follow-up access, catheter-care capacity, and clear rescue instructions. Virtual reality (VR) is another non-pharmacological adjunct that may be used for perioperative distraction, anxiety reduction, and rehabilitation engagement. A pediatric systematic review and meta-analysis reported that VR reduced patient-reported pain and anxiety during medical procedures, with standardized mean differences favoring VR for both pain and anxiety outcomes (108, 109). More directly, a randomized controlled trial of 99 school-age children undergoing limb fracture surgery found that both immersive and non-immersive VR gaming reduced perioperative pain, fear, and anxiety compared with conventional care, with immersive VR showing a more pronounced effect (110). These findings suggest that VR may be a useful adjunct for children with limb fractures, particularly when anxiety, fear, or poor cooperation interferes with perioperative care or early rehabilitation.

However, evidence that VR improves long-term fracture-specific functional outcomes, return-to-school timing, or return-to-sport timing remains limited, and these endpoints should be included in future pediatric orthopedic trials. Three-dimensional printing can be used to create patient-specific anatomical models for selected complex fractures, deformities, or growth-related orthopedic conditions, thereby supporting preoperative planning, simulation, and communication with families (111, 112). Quantitative fracture evidence supports this concept: in a study of 3D-printing-assisted treatment for old and complex acetabular fractures, the 3D-printing group had shorter operation time, less blood loss, and fewer fluoroscopy exposures than the conventional group (113). Because this evidence is not pediatric-specific, it should be used only as indirect support for the potential efficiency of 3D-assisted planning in selected complex pediatric orthopedic trauma cases. For children and caregivers, 3D-printed models may improve anatomical understanding, shared decision-making, and informed consent, although pediatric fracture-specific studies quantifying anxiety reduction or satisfaction remain scarce.

This enhanced understanding fosters cooperation and realistic expectations regarding recovery. The integration of continuous regional analgesia, VR-based distraction or rehabilitation, and 3D-assisted planning illustrates how specialized technologies may support selected components of pediatric orthopedic ERAS pathways. Overall, these technologies may improve pain control, perioperative experience, rehabilitation engagement, and surgical planning in selected pediatric orthopedic patients; nevertheless, future fracture-specific ERAS studies should report quantitative endpoints such as opioid use, pain scores, anxiety scores, rehabilitation adherence, operative time, fluoroscopy exposure, return-to-school or return-to-sport timing, complications, satisfaction, and cost.

## 6 Quantitative clinical outcome assessment and implementation challenges of ERAS protocols

**Figure 5:**
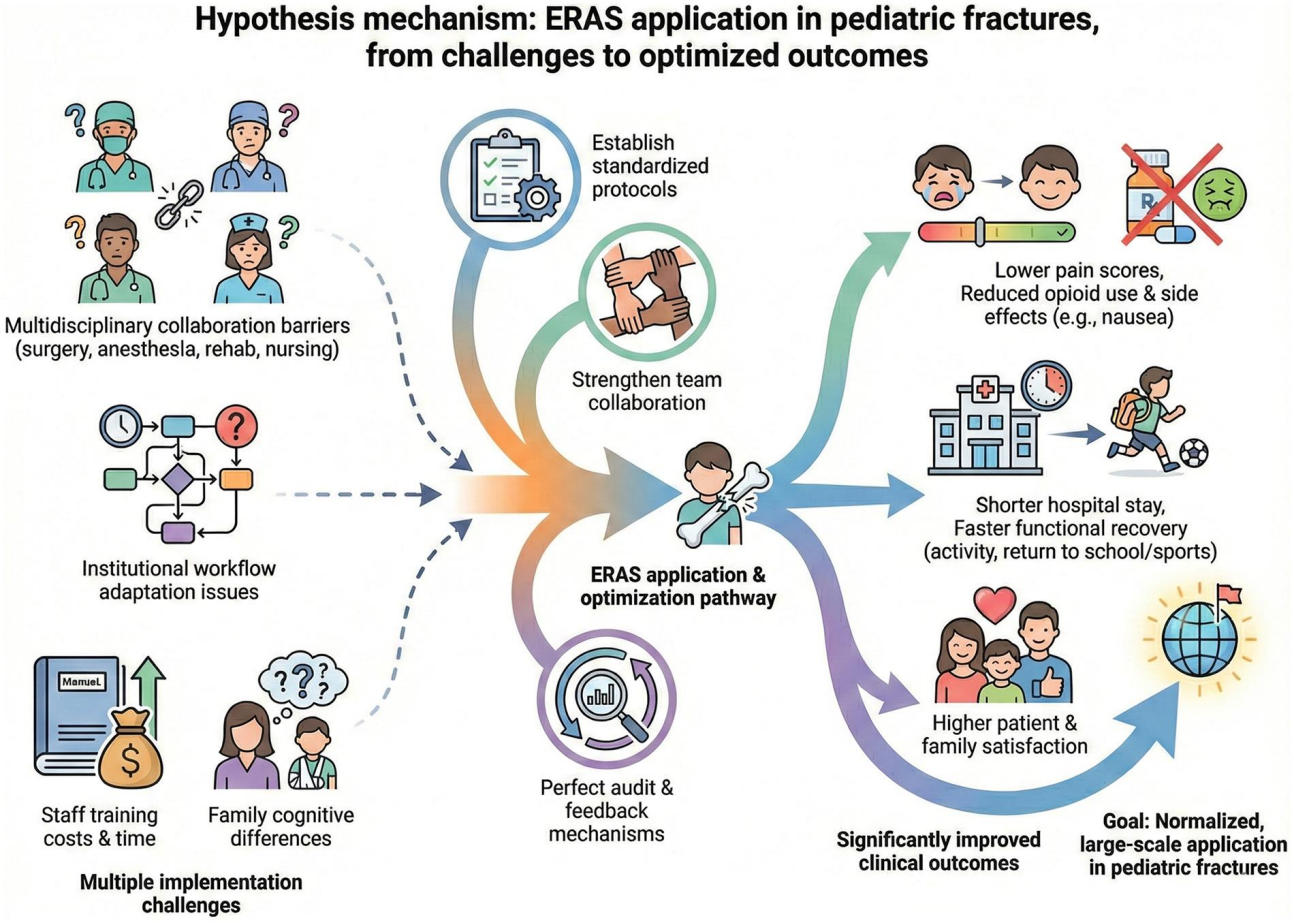
Hypothesized mechanism of ERAS application in pediatric fractures: from challenges to optimized outcomes. Multiple implementation challenges, including multidisciplinary collaboration barriers (surgery, anesthesia, rehabilitation, nursing), institutional workflow adaptation issues, staff training costs and time, and family cognitive differences, are addressed through a structured ERAS application and optimization pathway. This pathway involves three core strategies: (1) establishing standardized protocols; (2) strengthening interdisciplinary team collaboration; and (3) perfecting audit and feedback mechanisms. The resulting outcomes include lower pain scores, reduced opioid use and associated side effects (e.g., nausea), shorter hospital stays, faster functional recovery (activity, return to school/sports), and higher patient and family satisfaction, ultimately leading to significantly improved clinical outcomes and the goal of normalized, large-scale ERAS application in pediatric fracture care.

Although ERAS protocols have shown measurable benefits in several pediatric surgical and orthopedic settings, evidence specific to pediatric fracture and sports injury care remains limited and heterogeneous, making rigorous outcome evaluation and identification of implementation barriers essential. A structured outcome-assessment framework should include both objective clinical indices and patient- or caregiver-reported outcomes, so that the value of ERAS pathways can be evaluated using measurable and comparable endpoints. At the same time, institutional barriers, multidisciplinary coordination, staff training, family adherence, and data-collection capacity must be considered because they directly influence protocol fidelity and the reproducibility of clinical benefits. The conceptual mechanism by which ERAS-oriented care may translate implementation challenges into improved clinical outcomes through standardized protocols, multidisciplinary collaboration, and continuous quality improvement is visually outlined in Figure 5.

This section summarizes clinically meaningful outcome domains, available quantitative evidence, cost-related findings, and practical barriers to implementation, while emphasizing the need for standardized reporting in future pediatric fracture ERAS studies.

### 6.1 Quantitative patient-related and functional outcomes

In pediatric fractures and sports injuries, ERAS effectiveness should be assessed through a combination of clinical outcomes, functional milestones, and patient- or caregiver-reported outcome measures (PROMs) rather than relying on any single endpoint. Core short-term outcomes should include age-appropriate pain scores, cumulative opioid consumption, PONV incidence, time to oral intake, time to first mobilization, urinary catheter or drain duration where applicable, length of hospital stay, complications, readmissions, and early functional milestones such as independent ambulation and return to school. For instance, a retrospective cohort study of 100 children undergoing surgery for supracondylar humerus fractures compared an ERAS care group with a conventional care group at a 1:1 ratio and reported lower pain scores at postoperative days 3 and 7, shorter postoperative hospitalization, fewer complications, better Flynn functional scores, and higher nursing satisfaction in the ERAS group, although the study was retrospective and single-center (114).

At the broader pediatric ERAS level, a systematic review and meta-analysis including 16 studies and 1,723 children found that ERAS reduced postoperative opioid use by 0.86 morphine-equivalent mg/kg and was associated with earlier feeding and shorter hospital stay without increasing complications or readmissions (61). In adolescent idiopathic scoliosis surgery, a meta-analysis of 2,456 patients reported that ERAS was associated with first ambulation 29.6 h earlier, patient-controlled analgesia (PCA) discontinuation 0.53 days earlier, urinary catheter discontinuation 0.5 days earlier, and length of stay 1.6 days shorter, with similar complication and 30-day readmission rates (115). These findings support the clinical relevance of multimodal analgesia, early mobilization, and coordinated nursing care, but they should be interpreted as supportive evidence rather than definitive proof of efficacy across all pediatric fracture types. Secondary outcomes should include validated patient- and caregiver-reported measures, such as health-related quality of life, treatment satisfaction, pain interference, physical function, anxiety, fear of reinjury, parental anxiety, and caregiving burden.

Where feasible, standardized pediatric orthopedic PROMs, including PROMIS-based measures or other age-appropriate instruments, should be incorporated to improve comparability across studies and reduce subjective reporting bias (116, 117). These dimensions are particularly relevant in pediatrics, because caregiver understanding, home supervision, school reintegration, and rehabilitation adherence can substantially influence the child's recovery experience. For sports-related injuries, psychological readiness, fear of reinjury, and athlete identity should also be considered, because pediatric and adolescent athletes may experience anxiety, depressive symptoms, or delayed return-to-sport confidence after musculoskeletal injury (59, 60). Long-term outcomes are also necessary to evaluate whether early ERAS benefits translate into durable recovery, including fracture union, joint range of motion, muscle strength, refracture rate, return-to-school timing, return-to-sport timing, and psychological sequelae such as trauma-related anxiety or fear of reinjury.

However, long-term psychological and functional outcomes remain insufficiently reported in pediatric fracture ERAS studies, and future studies should prospectively measure these endpoints rather than simply describing psychological support as a theoretical component of care. Collectively, these outcomes provide a practical evaluation framework for pediatric orthopedic ERAS, but a standardized core outcome set is still needed to enable comparison across studies, procedures, institutions, and follow-up periods.

### 6.2 Healthcare system outcomes, cost implications, and implementation barriers

The implementation of ERAS-oriented pathways may affect healthcare-system outcomes, including resource utilization, cost-effectiveness, readmission rates, unplanned emergency visits, clinic-visit burden, staff workflow, and provider efficiency. Available pediatric orthopedic evidence suggests potential resource benefits, but the strength of evidence varies by procedure: for example, a before-after pediatric orthopedic study including 4,098 children reported improved organizational and financial indicators after interdisciplinary ERAS implementation, including a reduction in the average cost-to-revenue ratio from 82.70% to 72.16% (65). In adolescent idiopathic scoliosis (AIS) surgery, one medical and economic study reported that ERAS reduced mean hospital fees by 3,029 euros per patient and shortened mean length of stay from 6.5 days to 5 days without increasing complications (118). Another AIS study using a Team Integrated Enhanced Recovery protocol reported a 26% reduction in length of stay, a 7.9% reduction in total hospital costs, a 7.9% increase in daily contribution margins, and a 10.6% increase in daily net income (119).

In pediatric supracondylar humerus fractures, an ERAS nursing program was associated with shorter hospitalization and fewer complications, suggesting possible resource benefits; however, this study did not provide a formal cost-effectiveness analysis, so direct cost savings in fracture-specific ERAS care remain to be confirmed (114). Despite these potential benefits, the adoption of ERAS in pediatric trauma faces several practical and methodological barriers. A primary challenge is the need for close multidisciplinary collaboration among surgeons, anesthesiologists, nurses, rehabilitation therapists, nutritionists, mental health professionals, data managers, children, and caregivers, which requires institutional culture change, clear role allocation, and consistent communication. Initial costs associated with protocol development, staff training, digital infrastructure, patient education materials, and process standardization may hinder implementation, particularly in centers with limited pediatric orthopedic volume or limited rehabilitation resources.

Continuous education for healthcare providers and families is also essential, because inconsistent understanding of analgesic plans, mobilization restrictions, wound care, device management, and warning signs may reduce adherence and compromise recovery. To overcome these obstacles, audit and feedback mechanisms should be established from the beginning of pathway implementation. Such mechanisms should track both process adherence and outcomes, including preoperative education completion, regional anesthesia use, opioid consumption, time to oral intake, time to mobilization, catheter or drain duration, PONV, complications, readmissions, unscheduled visits, length of stay, family satisfaction, and direct medical costs. Continuous data collection on clinical outcomes, patient and caregiver satisfaction, process adherence, and cost indicators enables iterative quality improvement and helps determine which ERAS components provide measurable benefit in specific pediatric fracture subgroups.

For selected low-risk fracture pathways, virtual fracture clinic models may also reduce outpatient-visit burden and costs; a systematic review of pediatric virtual fracture clinics reported that all children were reviewed within 72 h, with limited missed fractures, re-presentation rates comparable to face-to-face clinics, significant cost savings, and high parent satisfaction (102). Nevertheless, virtual follow-up and mobile health tools should be limited to clearly defined, protocol-driven, low-risk cases and supported by safety-netting measures such as telephone access, rapid return pathways, and clear criteria for in-person reassessment. Overall, successful ERAS implementation in pediatric orthopedic trauma depends on multidisciplinary collaboration, institutional support, family engagement, standardized data collection, and continuous quality assurance; however, procedure-specific cost-effectiveness studies and prospective multicenter evaluations are still required before ERAS can be considered a fully validated standard for pediatric fracture care.

## 7 Literature search strategy and evidence selection

To improve methodological transparency and reduce potential selection bias, this narrative review was based on a structured literature search of PubMed/MEDLINE and Web of Science. The initial search covered publications available up to 22 February 2026, the date of manuscript submission. During revision, an updated search was performed to include newly available and relevant studies published up to July 2026, particularly those providing quantitative evidence regarding ERAS components and clinical outcomes. The main search terms included “enhanced recovery after surgery,” “ERAS,” “pediatric fracture,” “pediatric orthopedic trauma,” “sports injury,” “multimodal analgesia,” “regional anesthesia,” “early mobilization,” “early oral feeding,” “rehabilitation,” “telemedicine,” “digital health,” “cost-effectiveness,” and “healthcare resource utilization.” These terms were used alone and in combination to identify studies relevant to the application of ERAS principles in pediatric fractures, orthopedic trauma, and sports-related musculoskeletal injuries.

Studies were considered eligible if they addressed pediatric fractures, pediatric orthopedic trauma, sports-related injuries, perioperative ERAS strategies, multimodal analgesia, early mobilization, nutritional support, family-centered rehabilitation, digital health technologies, clinical outcomes, or healthcare-resource outcomes. Priority was given to studies directly involving pediatric orthopedic or pediatric surgical populations. When direct evidence in pediatric fracture care was limited, evidence from broader pediatric surgery, adult orthopedic ERAS, or general trauma care was included only when it was clinically relevant and cautiously applicable to pediatric orthopedic trauma. Articles were excluded if they were unrelated to ERAS principles or pediatric musculoskeletal injury care, focused exclusively on adult populations without reasonable translational relevance, or lacked sufficient clinical or methodological information to support the discussion.

This article was designed as a narrative review rather than a systematic review; therefore, no formal meta-analysis, Preferred Reporting Items for Systematic Reviews and Meta-Analyses (PRISMA)-based study selection process, or risk-of-bias assessment was performed. Nevertheless, we aimed to interpret the available evidence critically and to distinguish direct pediatric evidence from indirect or extrapolated evidence where appropriate. This approach was used to provide a clinically meaningful synthesis while acknowledging the heterogeneity of current studies and the limited availability of standardized ERAS protocols specifically designed for pediatric fracture and sports injury care.

## 8 Conclusion

The development of ERAS-oriented protocols for pediatric fractures and sports-related injuries represents a promising direction in perioperative care, reflecting a gradual shift toward patient-centered, evidence-informed, and recovery-focused management. The ERAS framework emphasizes multimodal strategies that address surgical stress, pain control, early mobilization, nutritional support, psychological needs, and family participation, thereby extending traditional fracture care beyond anatomical stabilization alone (62, 65, 120). This evolution underscores the importance of integrating clinical evidence, developmental considerations, and caregiver-centered support when designing recovery pathways for children undergoing orthopedic interventions (65).

Across the available evidence, a consistent theme is that ERAS implementation in pediatric orthopedic trauma requires a comprehensive, multidisciplinary, and family-centered approach (63, 65). Preoperative education is an important component because it may reduce anxiety, improve cooperation, clarify expectations regarding pain and rehabilitation, and strengthen family engagement in the recovery process (121, 122). Multimodal analgesia, combining non-opioid medications, regional anesthesia, opioid stewardship, and non-pharmacological support when appropriate, can improve pain control while reducing unnecessary opioid exposure, which is particularly important in pediatric care (123). Minimally invasive fixation techniques may reduce soft-tissue disruption and support earlier rehabilitation in appropriately selected pediatric fractures, although their contribution to ERAS outcomes should be distinguished from analgesia, mobilization, nutrition, and discharge planning (124, 125). Early rehabilitation protocols, when guided by fracture stability, pain control, age, and family capacity, may promote functional recovery and psychological well-being, highlighting the close relationship between physical recovery and emotional adaptation (126).

Digital health tools and specialized technologies may support ERAS implementation by improving follow-up continuity, rehabilitation adherence, symptom monitoring, analgesic delivery, and communication between clinicians and families (127–129). These innovations may enable remote monitoring, individualized care adjustment, and improved communication among multidisciplinary teams, but their fracture-specific effectiveness should be evaluated using measurable endpoints such as visit burden, complication detection, rehabilitation adherence, family satisfaction, and cost (129–131). However, the heterogeneity of existing studies—varying in design, patient populations, and outcome measures—necessitates cautious interpretation of results. Although available evidence suggests that ERAS pathways may reduce postoperative opioid use, shorten hospital stay, improve early mobilization, and support functional recovery in pediatric surgical or orthopedic populations, direct evidence in pediatric fracture-specific ERAS remains limited and requires further validation (61, 114, 115).

The economic implications of ERAS protocols are also clinically relevant, particularly for hospital leadership, resource allocation, and family burden. Available pediatric orthopedic evidence suggests potential cost and resource benefits, including reduced cost-to-revenue ratios, shorter hospitalization, and lower procedure-related hospital fees in some ERAS pathways, but these findings are mainly derived from broader pediatric orthopedic or adolescent scoliosis populations rather than pediatric fracture-specific cost-effectiveness studies (65, 118, 119, 132–134). Nevertheless, formal cost-effectiveness analyses tailored to pediatric fracture and sports injury ERAS are still scarce, and future studies should report direct medical costs, hospitalization costs, outpatient visit burden, readmissions, complications, caregiver time costs, and procedure-specific resource use.

Looking forward, the field should prioritize prospective, multicenter studies specifically designed for pediatric fracture and sports injury populations to refine and standardize ERAS-oriented protocols (26, 62). The development of specialty- and procedure-specific guidelines may facilitate more consistent implementation, outcome benchmarking, and comparison across institutions (26). Moreover, practical challenges such as multidisciplinary collaboration, staff training, family education, digital infrastructure, data collection, and individualized rehabilitation planning should be addressed during pathway design rather than after implementation. Addressing these barriers will require coordinated efforts from clinicians, rehabilitation teams, nurses, researchers, healthcare administrators, families, and policymakers.

Therefore, the present evidence should be interpreted as encouraging but not definitive, with stronger support for selected ERAS components and related pediatric orthopedic populations than for a fully validated, standardized ERAS pathway specific to all pediatric fracture types. Ultimately, the goal of ERAS in pediatric fracture and sports injury management extends beyond immediate postoperative recovery. By optimizing perioperative care through evidence-informed, child-centered, and family-supported strategies, ERAS has the potential to improve recovery experience and long-term quality of life for injured children. This holistic vision aligns with contemporary pediatric healthcare priorities that emphasize not only complication avoidance, but also pain relief, functional restoration, return to school or sports, psychological adaptation, and family satisfaction. As the evidence base grows and implementation strategies mature, ERAS-oriented care may become an increasingly important model for pediatric orthopedic trauma management, provided that future studies define standardized protocols, report quantitative outcomes, and clarify which components produce measurable benefits in specific fracture and sports injury populations.
